# Hematopoietic cell transplantation and cellular therapy survey of the EBMT: monitoring of activities and trends over 30 years

**DOI:** 10.1038/s41409-021-01227-8

**Published:** 2021-02-23

**Authors:** Jakob R. Passweg, Helen Baldomero, Christian Chabannon, Grzegorz W. Basak, Rafael de la Cámara, Selim Corbacioglu, Harry Dolstra, Rafael Duarte, Bertram Glass, Raffaella Greco, Arjan C. Lankester, Mohamad Mohty, Régis Peffault de Latour, John A. Snowden, Ibrahim Yakoub-Agha, Nicolaus Kröger, for the European Society for Blood and Marrow Transplantation (EBMT)

**Affiliations:** 1 EBMT Activity Survey Office, Division of Hematology, Department of Medicine, University Hospital, Basel, Switzerland; 2 Institut Paoli Calmettes Comprehensive Cancer Center & Inserm CBT-1409, Centre d’Investigations Cliniques en Biothérapies, Marseille, France; 3 Department of Hematology, Oncology and Internal Medicine, Medical University of Warsaw, Warsaw, Poland; 4 Department of Hematology, Hospital Universitario de la Princesa, Madrid, Spain; 5 Department of Pediatric Hematology, Oncology and Stem Cell Transplantation, University of Regensburg, Regensburg, Germany; 6 Laboratory of Hematology, Department of Laboratory Medicine, Radboud University Medical Center, Nijmegen, The Netherlands; 7 Servicio de Hematologia y Hemoterapia, Hospital Universitario Puerta de Hierro, Madrid, Spain; 8 Klinik für Hämatologie und Stammzelltransplantation, HELIOS Klinikum Berlin-Buch, Berlin, Germany; 9 Unit of Hematology and Bone Marrow Transplantation, IRCCS San Raffaele Scientific Institute, Vita-Salute San Raffaele University, Milan, Italy; 10 Willem-Alexander Children’s Hospital, Department of Pediatrics, Leiden University Medical Centre Leiden, Leiden, The Netherlands; 11 Department of Hematology, Hospital Saint Antoine, INSERM UMRs938, Sorbonne University, Paris, France; 12 BMT Unit, Department of Hematology, Hospital St. Louis, Paris, France; 13 Department of Haematology, Sheffield Teaching Hospitals NHS Foundation Trust, Sheffield, UK; 14 CHU de Lille, INSERM U1286, Infinite, Univ Lille, Lille, France; 15 Department of Stem Cell Transplantation, University Hospital Eppendorf, Hamburg, Germany

**Keywords:** Haematological cancer, Leukaemia

## Abstract

Numbers of Hematopoietic cell transplantation (HCT) in Europe and collaborating countries continues to rise with 48,512 HCT in 43,581 patients, comprising of 19,798 (41%) allogeneic and 28,714 (59%) autologous, reported by 700 centers in 51 countries during 2019. Main indications were myeloid malignancies 10,764 (25%), lymphoid malignancies 27,895 (64%), and nonmalignant disorders 3173 (7%). A marked growth in CAR-T cellular therapies from 151 in 2017 to 1134 patients in 2019 is observed. This year’s analyses focus on changes over 30 years. Since the first survey in 1990 where 143 centers reported 4234 HCT, the number has increased to 700 centers and 48,512 HCT. Transplants were reported in 20 countries in 1990, and 51, 30 years later. More than 800,000 HCT in 715,000 patients were reported overall. Next to the massive expansion of HCT technology, most notable developments include the success of unrelated donor and haploidentical HCT, an increase followed by decrease in the number of cord blood transplants, use of reduced intensity HCT in older patients, and the phenomenal rise in cellular therapy. This annual report of the European Society for Blood and Marrow Transplantation (EBMT) reflects current activity and highlights important trends vital for health care planning.

## Introduction

The European Society for Blood and Marrow Transplantation (EBMT) published for the first time in 1990 [1] a survey describing activity in hematopoietic stem cell transplant centers in Europe. Since then, this survey was published annually and herewith will be the 30th anniversary edition. The survey includes over 700,000 patients with over 800,000 transplants. Initially the survey was designed in the form of a single page spreadsheet for ease of reporting and has remained in this format ever since, although many additional features had been added, such as refined disease classification, information on conditioning intensity, pediatric activity, cell source, and cellular therapy.

Hematopoietic cell transplantation (HCT) is an established procedure for many acquired or inherited disorders of the hematopoietic system, benign or neoplastic, including those of the immune system, and as enzyme replacement in metabolic disorders [2–4]. The activity survey of the EBMT, describing the status of HCT, has become an instrument with which to observe trends and monitor changes in HCT technology in Europe and neighboring countries [5–14]. The survey, using a standardized structure, captures the numbers of HCT from highly committed participating centers, stratified by indication, donor type, and stem cell source. In the last few years, the survey has also included information on cellular therapies with hematopoietic cells for uses other than to replace the hematopoietic system [15–28, https://www.ema.europa.eu/en/documents/scientific-guideline/qualification-opinion-cellular-therapy-module-european-society-blood-marrow-transplantation-ebmt_en.pdf]. The analysis of the survey data since 1990 shows a continued and constant increase in the annual numbers of HCT and transplant rates for both allogeneic and autologous HCT. This report, based on the 2019 survey data, shows recent trends, changes in indications, and use in Europe and collaborating countries and summarizes the last 30 years.

## Patients and methods

### Data collection and validation

We invited participating centers to report their data for 2019 using the activity survey as shown in Table 1. The survey allows the reporting of additional information on the numbers of subsequent transplants performed due to relapse, rejection, or those that are part of a planned sequential protocol. Information on the numbers of patients receiving unmanipulated donor lymphocyte infusions (DLIs), non-myeloablative or reduced intensity HCT, and the numbers of pediatric HCT is also collected.

**Table 1:** Numbers of HCT in Europe 2019 by indication, donor type and stem cell source.

	Transplant activity 2019																	
	No. of patients																	
	Allogeneic												Autologous			Total		
	Family									Unrelated						Allo	Auto	Total
	HLA-id			Twin	Haplo ≥ 2MM		Other family						BM	BM+				
	BM	PBPC	Cord	All	BM	PBPC	BM	PBPC	Cord	BM	PBPC	Cord	Only	PBPC	Cord			
**Myeloid malignancies**	**294**	**2562**	**9**	**13**	**367**	**1359**	**14**	**73**	**0**	**417**	**5292**	**118**	**0**	**245**	**1**	**10,518**	**246**	**10,764**
Acute myeloid leukemia	227	1799	5	12	268	986	9	56	0	253	3305	87	0	237	0	7007	237	7244
1st Complete remission	144	1163	1	9	154	523	4	37	0	153	1852	38	0	193	0	4078	193	4271
Not 1st complete remission	58	427	4	3	74	327	4	16	0	72	848	35	0	41	0	1868	41	1909
AML therapy-related or myelodysplasia-related changes	25	209	0	0	40	136	1	3	0	28	605	14	0	3	0	1061	3	1064
Chronic myeloid leukemia	7	103	1	1	6	46	0	0	0	20	209	2	0	0	0	395	0	395
Chronic phase	3	54	1	0	2	13	0	0	0	7	91	2	0	0	0	173	0	173
Not chronic phase	4	49	0	1	4	33	0	0	0	13	118	0	0	0	0	222	0	222
MDS or MD/MPN overlap	52	475	3	0	64	259	4	14	0	130	1283	26	0	7	1	2310	8	2318
MPN	8	185	0	0	29	68	1	3	0	14	495	3	0	1	0	806	1	807
**Lymphoid malignancies**	**312**	**1321**	**8**	**6**	**207**	**841**	**9**	**51**	**1**	**346**	**2093**	**60**	**30**	**22,610**	**0**	**5255**	**22,640**	**27,895**
Acute lymphatic leukemia	267	745	6	0	124	478	7	34	1	286	1044	52	0	66	0	3044	66	3110
1st Complete remission	149	535	3	0	54	243	5	23	0	144	686	24	0	62	0	1866	62	1928
Not 1st complete remission	118	210	3	0	70	235	2	11	1	142	358	28	0	4	0	1178	4	1182
Chronic lymphocytic leukemia	4	40	0	0	6	19	0	0	0	3	99	0	0	11	0	171	11	182
Plasma cell disorders—MM	2	89	0	3	5	24	0	1	0	5	140	0	7	13,245	0	269	13,252	13,521
Plasma cell disorders—other	0	5	0	0	5	3	0	0	0	0	14	0	0	442	0	27	442	469
Hodgkin lymphoma	11	113	0	1	32	112	1	3	0	12	147	2	10	2175	0	434	2185	2619
Non Hodgkin lymphoma	28	329	2	2	35	205	1	13	0	40	649	6	13	6671	0	1310	6684	7994
**Solid tumors**	**3**	**2**	**0**	**0**	**3**	**14**	**0**	**0**	**0**	**2**	**4**	**1**	**18**	**1529**	**0**	**29**	**1547**	**1576**
Neuroblastoma	2	2	0	0	3	9	0	0	0	0	1	0	10	489	0	17	499	516
Soft tissue sarcoma/Ewing	1	0	0	0	0	2	0	0	0	0	1	1	2	245	0	5	247	252
Germinal tumors	0	0	0	0	0	0	0	0	0	0	1	0	2	431	0	1	433	434
Other solid tumors	0	0	0	0	0	3	0	0	0	2	1	0	4	364	0	6	368	374
**Nonmalignant disorders**	**731**	**343**	**25**	**7**	**123**	**205**	**65**	**55**	**1**	**530**	**461**	**58**	**7**	**561**	**1**	**2604**	**569**	**3173**
Bone marrow failure—SAA	185	140	0	5	21	54	5	10	0	181	138	10	0	1	1	749	2	751
Bone marrow failure—other	56	28	2	1	20	18	8	9	0	75	52	4	0	0	0	273	0	273
Thalassemia	154	45	9	0	2	11	17	9	1	42	52	0	1	0	0	342	1	343
Sickle cell disease	163	87	8	0	26	9	12	1	0	11	7	0	0	0	0	324	0	324
Primary Immune deficiencies	136	28	6	0	44	103	21	19	0	174	169	19	4	8	0	719	12	731
Inh. disorders of Metabolism	32	12	0	1	10	9	2	5	0	46	33	25	2	13	0	175	15	190
Autoimmune disease—MS	0	0	0	0	0	0	0	0	0	0	1	0	0	442	0	1	442	443
Autoimmune disease—SSC	0	0	0	0	0	0	0	0	0	0	0	0	0	55	0	0	55	55
Autoimmune disease—-other	5	3	0	0	0	1	0	2	0	1	9	0	0	42	0	21	42	63
**Others**	**21**	**14**	**0**	**0**	**11**	**17**	**5**	**6**	**0**	**36**	**42**	**5**	**0**	**16**	**0**	**157**	**16**	**173**
**Total patients**	**1361**	**4242**	**42**	**26**	**711**	**2436**	**93**	**185**	**2**	**1331**	**7892**	**242**	**55**	**24,961**	**2**	**18,563**	**25,018**	**43,581**
Re/additional transplants	36	155	0	2	59	332	2	11	1	68	547	22	5	3691	0	1235	3696	4931
**Total transplants**	**1397**	**4397**	**42**	**28**	**770**	**2768**	**95**	**196**	**3**	**1399**	**8439**	**264**	**60**	**28,652**	**2**	**19,798**	**28,714**	**48,512**

The bold numbers indicate the subtotals of the rows (i.e. the number of myeloid malignancies = the total of acute myeloid leukemia + chronic myeloid leukemia + MDS or MD/MPN overlap + MPN).

In addition, centers report information on different types of cellular therapies qualifying as advanced therapy medicinal products (ATMP) since they result from substantial manipulations of the collected cells, whether manufactured by industry centrally, or locally by an academic institution.

Quality control measures included several independent systems: confirmation of validity of the entered data by the reporting center, selective comparison of the survey data with MED-A data sets in the EBMT Registry database and crosschecking with National Registries.

### Centers

Since 1990, a directory of HCT centers consisting of both members of the EBMT and non members, in both European and collaborating non-European countries has been accrued. The directory is updated annually according to the centers current activity. In 2019, 730 centers from 53 countries were contacted (42 European and 11 collaborating countries); of which 700 centers responded. This corresponds to a 96% return rate and includes 82% of EBMT members. Thirty active centers failed to report in 2019. Reporting centers are listed in the Supplementary Online Appendix in alphabetical order, by country, city, and EBMT center code, with their reported numbers of first and total HCT, and of first allogeneic and autologous HCT. The WHO regional office definitions were used to classify countries as European or non-European. Ten collaborating non-European countries participated in the 2019 EBMT survey: Algeria, Iran, Iraq, Jordan, Lebanon, Nigeria, Saudi Arabia, South Africa, Syria, and Tunisia. Their data, 2590 HCT in 2502 patients, from 31 actively transplanting centers make up 5.3% of the total data set and are included in all analyses.

### Patient and transplant numbers

Wherever appropriate, patient numbers corresponding to the number of patients receiving a first transplant in 2019, and transplant numbers reflecting the total number of transplants performed are listed. The term sibling donor includes HLA identical siblings and twins but not siblings with HLA mismatches. Unrelated donor transplants include HCT from matched or mismatched unrelated donors with peripheral blood and bone marrow as a stem cell source but not cord blood HCT. Haploidentical transplants are being described as any family member with 2 or more (but not more than 5) loci mismatches within the loci HLA-A, -B, -C, -DRB1, and -DQB1 in GvH and/or HvG direction. Other family member donors are those related donors that are mismatched to a lesser degree than a full haplotype. For the purpose of analysis we add the small number of “other family donor” to haploidentical donor HCT. Additional non-first transplants may include multiple transplants defined as subsequent transplants within a planned double or triple autologous or allogeneic transplant protocol, and re-transplants (autologous or allogeneic) defined as unplanned HCT for rejection or relapse after a previous HCT.

### Hematopoietic advanced cellular therapies other than hematopoietic cell transplantation

Centers were requested to report all patients receiving cellular therapies other than HCT in 2019. Hematopoietic advanced cellular therapies were defined as infusion of cells undergoing substantial manipulation after collection, either selection and/or expansion, or genetic modification and thus qualify as investigational or approved ATMPs according to Regulation (EC) N° 1394/2007. In this context, “substantial” should be understood as referring to the definition included in the Regulation and subsequent regulatory documents and may not reflect the workload assumed by cell processing facilities working in conjunction with clinical programs. Depending on their nature and indications, hematopoietic cellular therapies may be designed to replace or to complement HCT. Administration of non-substantially manipulated hematopoietic cells, such as transplantation of CD34+ selected hematopoietic stem cells is counted as HCT and not as cellular therapy [15]. Similarly, unmanipulated lymphocyte infusions post-HCT are counted as DLIs and not cellular therapy. Hematopoietic cellular therapies include immune effector cells as defined in FACT-JACIE standards for Hematopoietic Cellular Therapy: “A cell that has differentiated into a form capable of modulating or effecting a specific immune response” [25, 26]. This definition covers CAR-T cells and forms the basis for accreditation requirements in recent EBMT-JACIE recommendations [16].

Hematopoietic cellular therapies were categorized as chimeric antigen receptor T-cells (CAR -T); in vitro selected/and or expanded T-cells or cytokine activated, such as virus specific T-cells; cytokine-induced killer cells (CIK); regulatory T cells (TREGS); genetically modified T cells other than CAR-T; natural killer cells; dendritic cells; mesenchymal stromal cells; in vitro expanded CD34+ cells; and genetically modified CD34+ cells. This survey does not include cells from sources other than hematopoietic tissue [19]. On the other hand, gene therapy protocols, such as those used to treat thalassemia or SCID are part of this survey, but currently numbers are low.

### Transplant and cellular therapy rates

Transplant rate, defined as the total number of HCT per 10 million inhabitants were computed for each country (based on the center report), without adjusting for patients receiving their HCT in a foreign country. Cellular therapy rates are defined as the numbers of patients receiving a cellular therapy treatment per 10 million population. Center density is defined as the number of centers performing a certain type of procedure per 10 million population. Population numbers for the European countries in 2019 were obtained from Eurostats: (http://appsso.eurostat.ec.europa.eu) and the World Bank database for the non-European countries: (https://databank.worldbank.org).

### Analysis

Wherever appropriate, the absolute numbers of transplanted patients, transplants or transplant rates are shown for specific countries, indications, or transplant techniques. Myeloid malignancy includes acute myeloid leukemia (AML), myelodysplastic or myelodysplastic/myeloproliferative neoplasia (MDS or MDS/MPN overlap), myeloproliferative neoplasm (MPN), and chronic myeloid leukemia (CML). Lymphoid malignancy includes acute lymphocytic leukemia (ALL), chronic lymphocytic leukemia (CLL), Hodgkin lymphoma (HL), non-Hodgkin lymphoma (NHL), and plasma cell disorders (PCD) (which includes multiple myeloma (MM) and others). The nonmalignant disorders include bone marrow failure (BMF (which includes severe aplastic anemia (SAA) and others), thalassemia and sickle cell disease (HG), primary immune disease (PID), inherited diseases of metabolism (IDM), and autoimmune diseases (AID). Others include histiocytosis and other rare disorders not included in the above.

## Results

### Participating centers in 2019

Of the 700 centers, 451 (64%) performed both allogeneic and autologous transplants; 229 (33%) restricted their activity to autologous HCT, and 18 (3%) to allogeneic transplants only. Two of the 700 responding centers reported no activity due to renovation or changes within the transplant unit. Within the 698 actively transplanting centers in 2019, 128 (18%) performed transplants on both adult and pediatric patients. An additional 122 (18%) were dedicated pediatric transplant centers and 448 (64%) perform transplants on adults only. Thirty centers failed to report in 2019, which, when compared with previously reported data, accounts for ~780 non-reported HCTs.

### Numbers of patients, transplants, and trends in 2019

In 2019, 48,512 transplants were reported in 43,581 patients (first transplant); of these, 19,798 HCT (41%) were allogeneic and 28,714 (59%) autologous (Table 1). Compared with 2018, the total number of transplants increased by 2.2% (0.9% allogeneic HCT and 3.1% autologous HCT) [13]. The corresponding number of patients showed an increase of 0.4% for allogeneic HCT and 2.5% for autologous HCT. In addition, there were 4931 s or subsequent transplants, 1235 being allogeneic, mainly to treat relapse or graft failure and 3696 autologous, the majority of which were likely to have been part of multiple transplant procedures such as tandem procedures, or as salvage autologous transplants for PCD. Furthermore, 819 of the allogeneic HCTs were reported as being given after a previous autologous HCT and were mainly for lymphoma or PCD.

The number of pediatric patients (<18 years old at transplant) transplanted in both dedicated pediatric and joint adult-pediatric units was 5189 (3990 allogeneic and 1199 autologous). This is an overall decrease of 3.3% in the total number of transplants; 2.1% allogeneic and 7.3% autologous HCT when compared to 2018. Of these, 3987 patients, (3123 allogeneic (78%), and 864 autologous (22%)) were treated in 121 dedicated pediatric centers in 27 countries. Due to the design of the survey, detailed analysis is limited in the dedicated centers only. Main indications for allogeneic HCT were AML (*n* = 414; 70% in early stage), ALL (*n* = 766; 45% in early stage), and NMD (*n* = 1534; 39% PID). There were 1625 family and 1498 unrelated donor HCTs reported. Within family donors, 42% were from a haploidentical relative. Bone marrow was used as the stem cell source in 1628 patients of which 56% were family donors. Peripheral blood stem cells were used in 1376 patients with equal proportions seen in both family (*n* = 685) and unrelated donors (*n* = 691). Cord blood stem cells were used in 119 patients of which 75% were unrelated HCT. The main indications for autologous HCT, were solid tumors, with 647 HCT reported in 2019, primarily for neuroblastoma (49%).

### Main indications

**Fig. 1:**
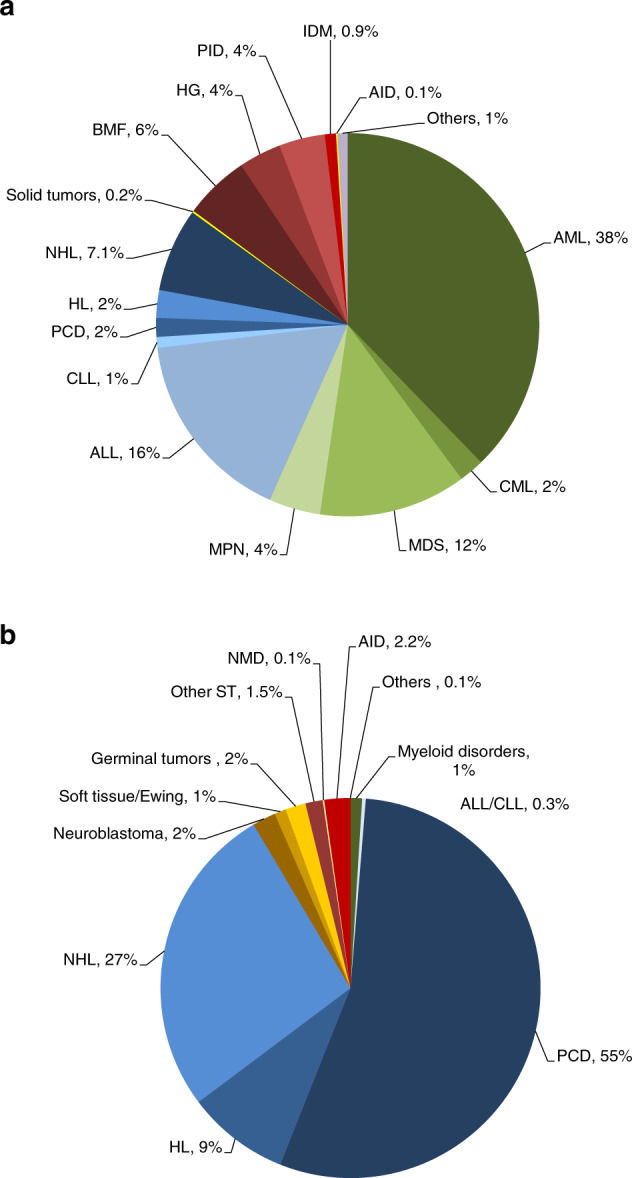
Relative proportion of disease indications for HCT in Europe 2019. **a** Relative proportion of allogeneic HCT. **b** Relative proportion of autologous HCT.

Indications for HCT in 2019 are listed in detail in Table 1 (Fig. 1a, b show distribution of disease indications for allogeneic (Fig. 1a) and autologous (Fig. 1b) HCT). Main indications for allogeneic HCT were myeloid malignancies (AML, CML, MDS or MDS/MPN overlap and MPN): 10,518 (98% allogeneic HCT and 2% autologous HCT). For autologous HCT, the main indications were lymphoid malignancies (ALL, CLL, PCD, HL, and NHL): 22,640 (19% allogeneic HCT and 81% autologous HCT).

### Allogeneic HCT

The leading indication for allogeneic HCT was AML, which accounts for 38% of all allogeneic HCT, an increase of 1% when compared to 2018. Increases were seen in both early disease stage (1.7%) and therapy-related AML or those with myelodysplasia-related changes (3.5%). AML in late disease stage decreased by 2.2%. Among the myeloid malignancies, CML continues to increase overall by 6.2%. However, differences were seen in 1st chronic phase with a decrease of 14.4% and advanced phase which increased by 30.6%, although overall the numbers remain low (*n* = 395 patients). Allogeneic HCT for MDS decreased slightly by 0.5% to 2310 patients treated. MPN increased by 7.8% from 748 in 2018, to 806. ALL comprises 16.4% of allogeneic HCT and showed a slight increase overall of 2.7% compared to the previous year. Again, differences were seen between early stage, increasing by 4.8% and late stage, decreasing by 0.6%. Allogeneic HCT for CLL continued to decrease by 10.9% when compared to 2018, a constant trend over recent years. Allogeneic HCT for NHL decreased by 4.1% to 1310 while for HL, rates remained stable. Within the nonmalignant disorders, a continued increase of 3.7% is seen for BMF—SAA (*n* = 749), and of 17.7% for BMF—non SAA (*n* = 273). PID increased by 6.4% (*n* = 719) and sickle cell disease by 44% (*n* = 324). For IDM, the rate decreased by 11.6% (*n* = 175) and for thalassemia by 17.4% (*n* = 342). Allogeneic HCT for AID remain a rare indication with just 22 patients treated in 2019, although there is an overlap with PID and other inherited disorders that would benefit from better definition for future reporting [27]. Within allogeneic HCT, 7625 were performed using non-myeloablative or reduced intensity conditioning in 2019. This comprises 39% of all allogeneic HCTs, a rate that has remained stable over the last 10 years.

### Autologous HCT

The main indications for autologous HCT were lymphoid malignancies (90%) with PCD comprising 55% of all autologous HCT patients. Autologous HCT for NHL has not changed over time up to 2019 while PCD have increased slightly by 4.2%. As reported in 2018, AML continued to decrease by 19.1% and ALL by 10.8%. For solid tumors and AID, the numbers remained stable. In 2019, the activity survey was adapted to capture more defined data for AID. The majority were performed for multiple sclerosis (*n* = 442), followed by systemic sclerosis (*n* = 55) and other AID (*n* = 42).

### Transplant rates and center density

**Fig. 2:**
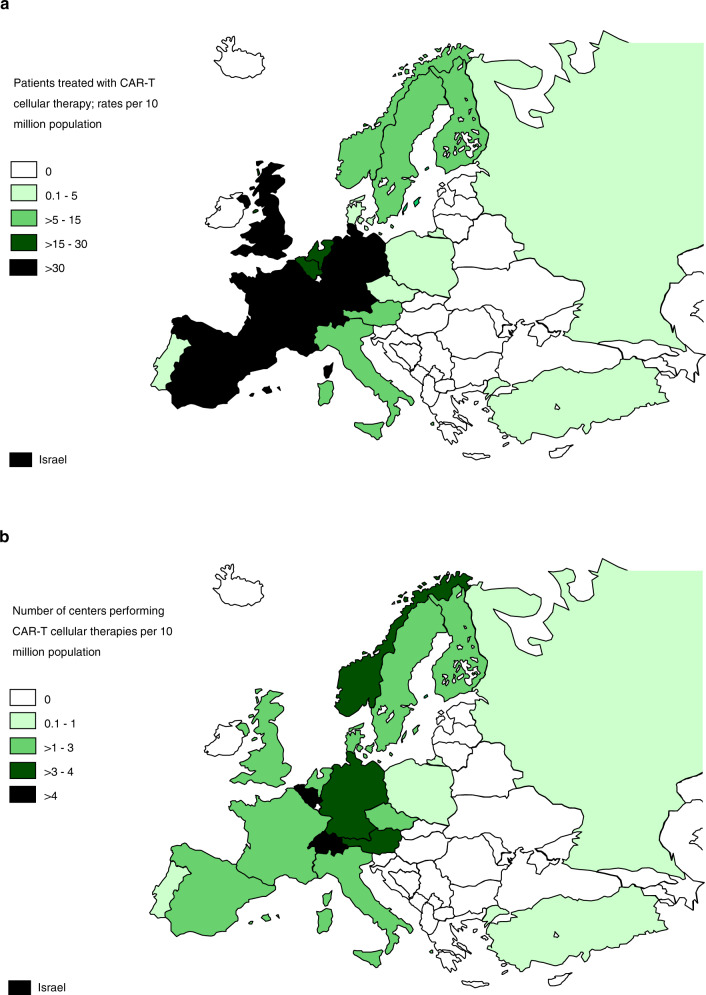

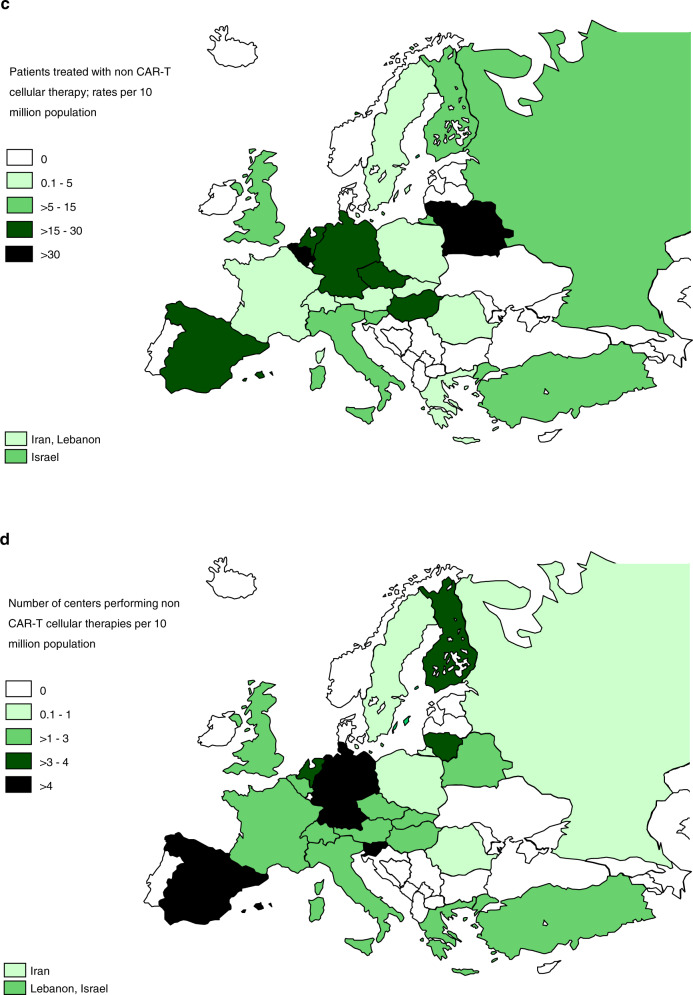
Continued.

Assessing transplant rates per 10 million population (TR) allows the comparison of activity in countries where population numbers differ greatly. Center density per 10 million population allows the comparison of activity by the number of centers. The TR rates for allogeneic HCT, ranged from 0.2 in Nigeria to 476.1 in Israel (median number of HCT 127 and TR 144) (Supplementary Fig. 1a). Five countries did not report any allogeneic HCT (Armenia, Bosnia and Herzegovina, Cyprus, Iceland, and Luxembourg). For autologous HCT, rates ranged from 2.3 in Syria to 625 in Switzerland (median number of HCT 181 and TR 281), (Supplementary Fig. 1b). Figure 2a shows the use of CAR-T cells in 2019 on a map as  treatment rates and (Fig. 2b) as center density. Figure 2c, d correspondingly depict non-CAR-T cellular therapies as treatment rates and as center density respectively.

### Donor type and stem cell source

In 2019, the overall numbers of patients treated with family donors remained stable, however, variation was seen within the choice of family donor used. HLA identical sibling and syngeneic twin donors decreased by 6% when compared to 2018 but increases were observed in haploidentical donors of 11% and unrelated donors of 1.2%. The cord blood HCT rate continued to decrease slowly (1%) and mainly included unrelated cord blood (85%). In sibling donors, peripheral blood and bone marrow stem cell use decreased by 6.2% and 5.6% respectively. In haploidentical donors, an increase of 17.8% was seen in the use of stem cells harvested from peripheral blood while use of bone marrow stem cells decreased by 6.8%. In unrelated donor transplants, the use of bone marrow and cord blood stem cells has decreased by 4.5% and 4%, respectively. For allogeneic HCT, the numbers increased only slightly (0.4%) and the proportion of those using bone marrow decreased by 5.4%. When compared to 2018, the absolute numbers of patients treated with autologous HCT has continued to increase (2.5%) and since 1996 are predominantly performed using peripheral blood stem cells (>90%).

### Thirty-year overview

The first activity survey was conducted in 1990 and presented data on 4234 patients reported by 143 centers in 21 countries. Since this first survey, revisions were made to ensure that each annual survey was optimized for the current changes in technology and treatment protocols used at the time. The survey allows us to monitor activities and trends over a 30-year period. The number of reporting centers has increased from 143 to 700 in 2019, and from 20 countries to 51. Allogeneic HCT has increased from 2137 to 19,798, while autologous HCT has increased from 2097 to 28,714.

**Fig. 3:**
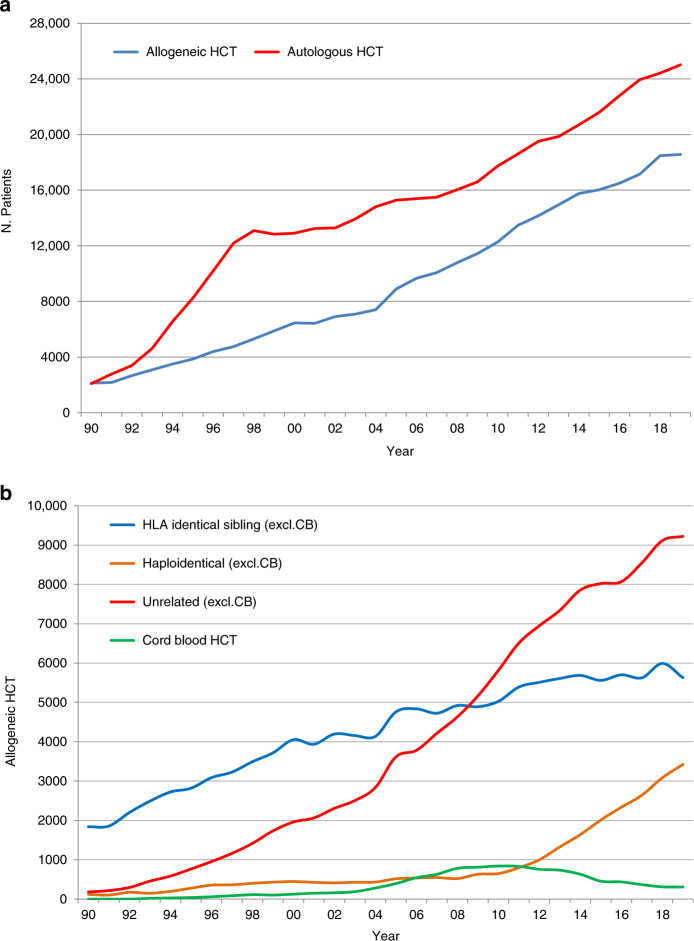
Development of HCT from 1990 to 2019. **a** Number of patients receiving autologous and allogeneic HCT. **b** Distribution of donor type among allogeneic HCT recipients.

**Fig. 4:**
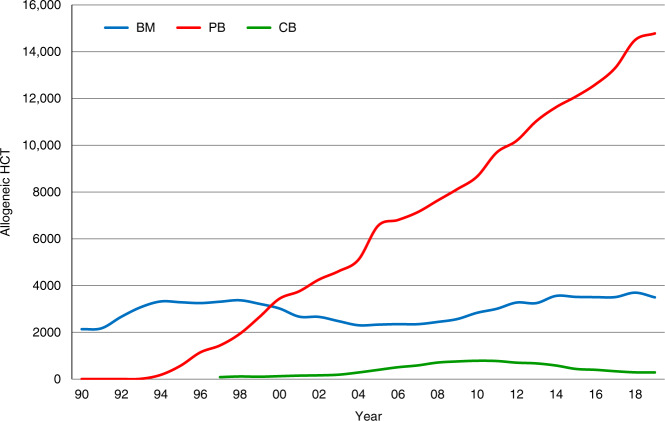
Change in choice of stem cell source for allogeneic HCT from 1990 to 2019. Figure shows the change in the use of bone marrow (BM), peripheral blood (PB) and cord blood (CB) as stem cell source over the 30 year period.

**Fig. 5:**
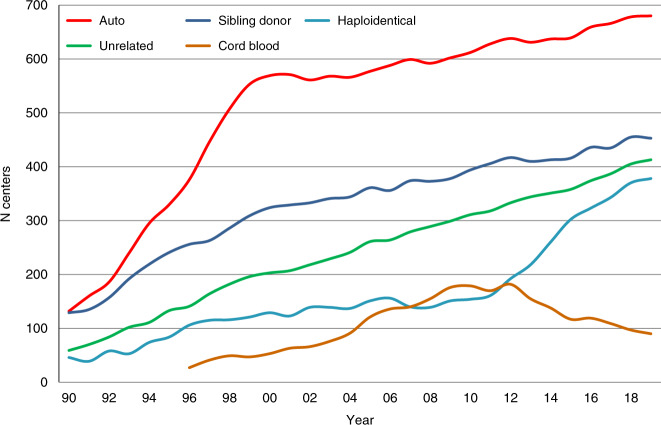
Change in choice of donor type by center from 1990 to 2019. The figure shows the numbers of centers and the type of donors selected for HCT over the 30 year period.

Figure 3a shows the development in the numbers of patients treated with autologous and allogeneic HCT over the 30-year period. Figure 3b shows the distribution of donor type among allogeneic HCT recipients depicting the importance of unrelated donor and haploidentical donor HCT globally in the recent decade. Figure 4 shows the changes in choice of stem cell source for allogeneic HCT. Autologous HCT does not warrant graphical depiction as the use of bone marrow has almost disappeared. It is evident that for allogeneic HCT, growth is due to the use of peripheral blood stem cells; use of marrow remains stable at around 3500 transplants annually and is prevalent in allogeneic HCTs for nonmalignant disorders (Table 1). Figure 5 addresses the number of centers performing HCT in Europe, increasing from 143 to 700 over 30 years. The Figure shows the number of centers performing autologous and allogeneic HCT using sibling, unrelated and haploidentical donors as well as cord blood. The number of centers performing autologous HCT rose sharply in the late 90s, most likely associated with its use in the treatment of solid tumors. Regarding allogeneic HCT, at all points in time the number of centers performing transplants with donors other than HLA identical siblings was lower than the number of centers performing sibling transplants. Since around 2015, there appears to be a convergence of centers performing allogeneic HCT using all types of available donor. Since 2012, the number of centers using cord blood donors as the stem cell source has declined.

### Cellular therapy

Table 2 shows the number of patients receiving advanced cellular therapy and DLI performed in 2019. There were 3028 patients receiving unmanipulated DLIs, which is a slight decrease of 2.2% since 2018. The majority of DLIs were given for relapse (*n* = 1461) and graft enhancement/failure (*n* = 716).

**Table 2:** Numbers of patients treated with a cellular therapy in Europe 2019 by indication, donor type and cell source.

Number of patients	DLI	CART		MSC		NK cells		Selected/expanded T cells or CIK		Regulatory T cells (TREGS)		Genetically modified T cells		Dendritic cells		Expanded CD34+ cells		Genetically modified CD34+ cells		Other		*Total excl DLI*	
2019		Allo	Auto	Allo	Auto	Allo	Auto	Allo	Auto	Allo	Auto	Allo	Auto	Allo	Auto	Allo	Auto	Allo	Auto	Allo	Auto	*allo*	*auto*
GvHD				296		1		1		48										9	1	355	1
Graft enhancement				20		1		22								7	1	1		173	27	224	28
Autoimmune dis.				24	8										3				4		3	24	18
Genetic disease				1															14	1	2	2	16
Infection				4				203	9	1		5	1							37		250	10
Malignancy—ALL		20	232	4		2		4				4			6					2	1	36	239
Malignancy—HL/NHL			826			1		3													4	4	830
Malignancy—other		1	55	1	1	8		14	21	13		2	1		25				4	20	11	59	118
DLI for graft enhancement/failure	716																					0	0
DLI for residual disease	431																					0	0
DLI for relapse	1461																					0	0
DLI per protocol	420																					0	0
**Total**	**3028**	**21**	**1113**	**350**	**9**	**13**	**0**	**247**	**30**	**62**	**0**	**11**	**2**	**0**	**34**	**7**	**1**	**1**	**22**	**242**	**49**	**954**	**1260**

**Fig. 6:**
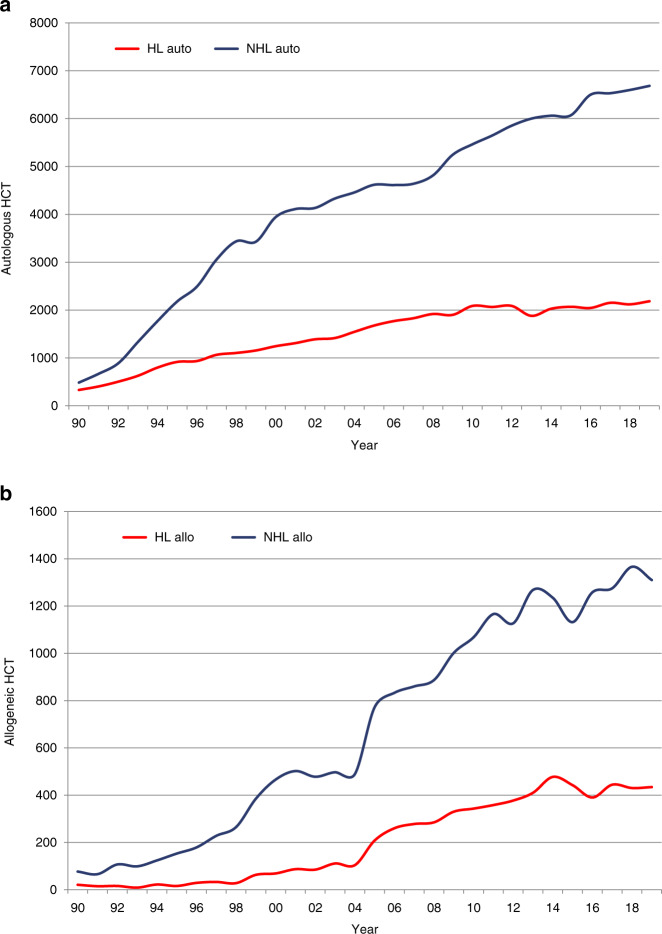
Change in HCT for lymphomas 1990–2019. **a** Autologous HCT for Hodgkin and non-Hodgkin lymphoma. **b** Allogeneic HCT for Hodgkin and non-Hodgkin lymphoma.

A total of 2214 patients in 198 centers from 31 countries received other forms of hematopoietic cellular therapies that qualify as medicinal products rather than cell transplants [15]. In 2019 the most remarkable increase was in gene-modified T cells, notably CAR-T cells increasing from 151 in 2017 to 1134 in 2019 (650% increase). One hundred and fifteen centers in 2019 in 19 countries reported CAR-T cellular therapies (Fig. 2a shows rates of CAR-T-cell treatment and Fig. 2b, center density performing CAR-T treatment). The main indication being lymphoma (*n* = 826; 100% autologous), followed by ALL (*n* = 252; 92% autologous), and other malignancies (*n* = 56; 98% autologous). Much has been written about CAR-T cellular therapy replacing autologous or allogeneic HCT for lymphoma, Fig. 6 shows numbers of autologous (Fig. 6a) and allogeneic (Fig. 6b) HCT for Non-Hodgkin lymphoma and Hodgkin lymphoma over 30 years. At this point in time it is too early to predict whether there has been an impact of CAR-T cell use on HCT. If the more than 800 CAR-T treatments for lymphoma had replaced HCT use, a notable decrease in activity would have been expected. The second most widely used cellular therapy other than CAR-T cells in 2019 is mesenchymal stromal cells (*n* = 359; 97% allogeneic), their use being mainly to treat graft-versus-host disease [19]. Since 2018, an increase was also seen in the numbers of selected/expanded T cells or CIK (127%) and TREGS (59%). Figure 2c shows rates of non-CAR-T-cell treatment and Fig. 2d, center density performing non-CAR-T treatment.

## Discussion

The EBMT activity survey has been conducted annually since 1990 [1]. Over 48,000 transplants in more than 43,500 patients were reported in 2019. Starting in 2015, we observed a slower growth for unrelated donor HCT as compared to haploidentical HCT [12]. In the last year, however, use of both types of donor continues to increase; by 10% for haploidentical donors, and 1.9% for unrelated donors. It is mostly in well-established indications where growth is observed, such as allogeneic HCT for AML in CR1, but also ALL in CR1, MPN, and PID.

In the 2017 report we described lower rates of aplastic anemia transplantation possibly due to the use of thrombopoietin analogues such as eltrombopag. In the last 2 years though, we see the number has increased by 28.6%, possibly suggesting that transplants are now performed later after failing thrombopoietin analogues but also centers proceeding quicker to transplantation as well as the growing upfront approach for children and younger adults.

The most impressive growth is observed in hematopoietic cellular therapies, most notably in the use of CAR-T cells, increasing to 1134 reported patients treated in 2019. Since the only two approved products received a centralized marketing approval from EMA in August 2018, it is possible that the reported activity in 2019 may still partially reflect patients in clinical studies, either industry-sponsored or academia sponsored [https://www.ema.europa.eu/en/documents/scientific-guideline/qualification-opinion-cellular-therapy-module-european-society-blood-marrow-transplantation-ebmt_en.pdf]. Investigational CAR-T cells can be produced by academic facilities. Since the EMA approved the first CAR-T cell product, a notable increase in the use of CAR-T cells has been observed and a further increase is to be expected in 2020 [22, 23]. Emergence of a rapidly growing clinical activity is reassuring in view of earlier reports demonstrating that Europe lagged behind the USA and China [24]. Figure 2b illustrates wide variability in center density for CAR-T cell treatment. Obviously, it is difficult to state an optimal number of centers for a given population at this point in time. The survey will continue to observe this development. The constant and steady increase in autologous or allogeneic HCT for NHL has not changed over time. These are indications for autologous as well as for allogeneic HCT which may in the future be replaced by CAR-T treatments. So far, there is little evidence for a replacement of transplant technology by cellular therapy products. While the use of transplant technology for autologous HCT for PCD continues to increase, the use of allogeneic HCT for PCD is decreasing; this may be due to the availability of more potent drugs such as monoclonal antibodies. The 30-year comparison shows the impressive expansion of the use of HCT technology across Europe. Whereas one can still criticize a lack of standardization in transplant indications and technologies, continued growth in established indications hints at a convergence of practices across Europe. Of interest is the use of HCT using alternative donors in Europe where centers specializing in certain types of transplants have given way to centers more commonly using the entire spectrum of sibling, unrelated donor and haploidentical donor transplantation reflecting an increased choice of donor available for patients in need of allogeneic HCT.

The annual activity survey of the EBMT reflects current activity and trends in the field of transplant technology. It is valuable for the dissemination of the most recent information on indications, donor and stem cell usage, and benchmarking of data completeness and survival outcomes [28], which will ultimately be beneficial in health care planning.

## Supplementary information

Appendix: list of reporting transplant centers participating in the 2019 activity survey

Supplementary figures 1a 1b
