# Supplementary figures and images for: Hematopoietic cell transplantation and cellular therapy survey of the EBMT: monitoring of activities and trends over 30 years
Source: Bone Marrow Transplant. 2021 Feb 23;56(7):1651–64. doi: 10.1038/s41409-021-01227-8 (PMC8263343; doi:10.1038/s41409-021-01227-8)

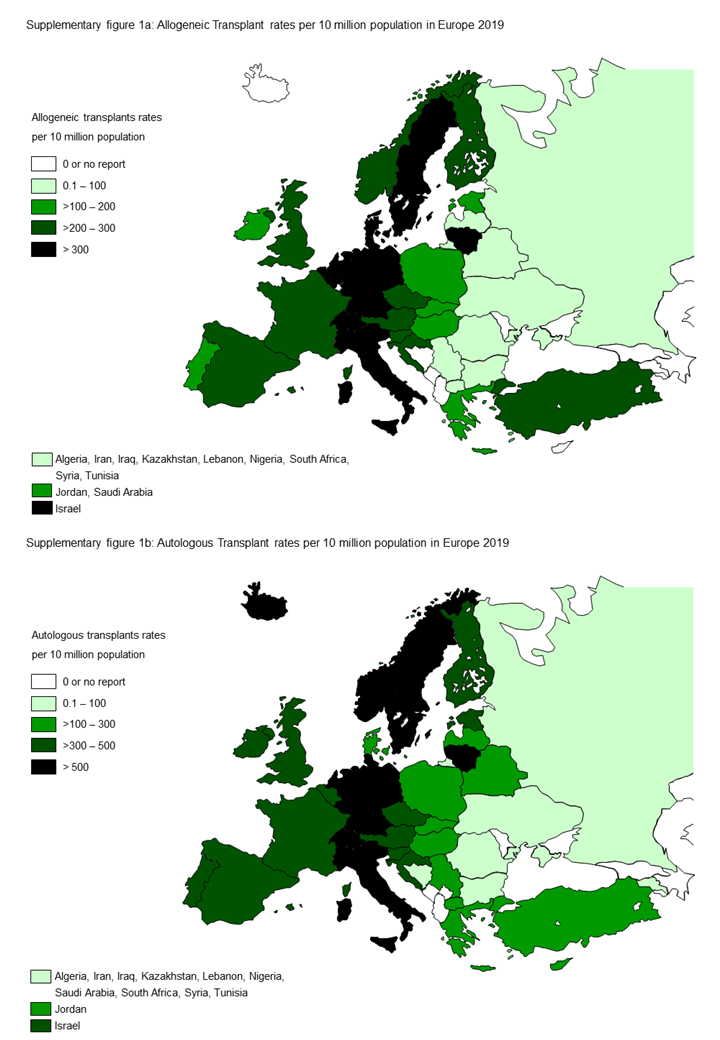

Supplement: Supplementary file 2 — Supplementary figures 1a 1b [file 41409_2021_1227_MOESM2_ESM.tif]
